# Primary cilia in Parkinson’s disease: summative roles in signaling pathways, genes, defective mitochondrial function, and substantia nigra dopaminergic neurons

**DOI:** 10.3389/fnagi.2024.1451655

**Published:** 2024-09-18

**Authors:** Zijiao Tian, Yixin Zhang, Jing Xu, Qianwen Yang, Die Hu, Jing Feng, Cong Gai

**Affiliations:** ^1^Dongzhimen Hospital, Beijing University of Chinese Medicine, Beijing, China; ^2^College of Acupuncture and Massage, Beijing University of Chinese Medicine, Beijing, China; ^3^Dongfang Hospital, Beijing University of Chinese Medicine, Beijing, China; ^4^College of Traditional Chinese Medicine, Beijing University of Chinese Medicine, Beijing, China

**Keywords:** primary cilia, Parkinson’s disease, signaling pathway, gene, defective mitochondrial function, substantia nigra dopaminergic neuron

## Abstract

Primary cilia (PC) are microtubules-based, independent antennal-like sensory organelles, that are seen in most vertebrate cells of different types, including astrocytes and neurons. They send signals to cells to control many physiological and cellular processes by detecting changes in the extracellular environment. Parkinson’s disease (PD), a neurodegenerative disease that progresses over time, is primarily caused by a gradual degradation of the dopaminergic pathway in the striatum nigra, which results in a large loss of neurons in the substantia nigra compact (SNpc) and a depletion of dopamine (DA). PD samples have abnormalities in the structure and function of PC. The alterations contribute to the cause, development, and recovery of PD via influencing signaling pathways (SHH, Wnt, Notch-1, α-syn, and TGFβ), genes (MYH10 and LRRK2), defective mitochondrial function, and substantia nigra dopaminergic neurons. Thus, restoring the normal structure and physiological function of PC and neurons in the brain are effective treatment for PD. This review summarizes the function of PC in neurodegenerative diseases and explores the pathological mechanisms caused by PC alterations in PD, in order to provide references and ideas for future research.

## 1 Introduction

Parkinson’s disease (PD) is a progressive neurodegenerative disorder (Jagadeesan et al., 2017). Since the 1990s, there have exceeded 7 million patients with PD worldwide. This figure is anticipated to increase as the global population ages and life expectancy increases (Rajan and Kaas, 2022). PD has become a common neurological disease in the 20th century, with recent data showing that there are at least a million patients with PD in the US, spending about $52 billion a year on treatment, and Chinese citizens with more than 3.6 million PD (Grotewold and Albin, 2024). The cardinal manifestations of PD include rigidity, slowness of movement, postural instability, gait dysfunction, rest tremors, and a variety of other dysfunctions of the tomotor and non-motor functions (Videnovic and Golombek, 2013; Capriotti and Terzakis, 2016). The underlying cause of PD is the progressively worsening dopaminergic pathway in the striatum nigra, which results in a significant loss of neurons in the substantia nigra compact (SNpc) and a deficiency of dopamine (DA) (Weintraub et al., 2022). Emerging studies suggest that both the growth of the brain and brain disorders depend heavily on PC (Park et al., 2019), and the pathology of PD is associated with defective PC (Gazea et al., 2016).

PC are microtubules-based, independent antennal-like sensory organelles, that are seen in most vertebrate cells of different types (Tereshko et al., 2022). The PC structure consists of basal bodies, transition regions, and axons (Ma et al., 2021). Microtubules are surrounded by the lateral ciliary membrane attached to the cell membrane (Mill et al., 2023). A large number of receptors involved in signal transduction exist on the ciliary membrane, such as Sonic hedgehog (SHH), WNT, and Notch (Patel and Smith, 2023). The basal body consists of several Bardet-Biedl syndrome (BBS) proteins, whose BBS proteins are essential for PC transport. And ciliary transport is regulated by intrafylagellar transport (IFT) (Lacey et al., 2023; Tian et al., 2023; Yeo et al., 2023; DeMars et al., 2023). The transition zone (TZ) of the ciliary base controls the lateral diffusion of membrane proteins between the cell body and the cilia (Zaidi et al., 2022; Gopalakrishnan et al., 2023). PC are able to detect changes in the extracellular environment and transmit signaling information to cells to regulate diverse cellular, developmental, and physiological processes (Silva and Cavadas, 2023). Thus, ciliary damage can lead to dysregulated signaling, leading to ciliopathy, which affects most of the tissues or organs of the body.

Newborn neurons rely on PC for synapsis; deficiencies in PC cause shorter neuronal dendrites and impaired neural integration into the adult brain, triggering a series of degenerative diseases (such as PD, AD, etc.) (Kumamoto et al., 2012). It has been shown that an important factor in the formation of PC is cellular senescence, and that the increased number and length of PC are associated with decreased cell proliferation (Zhao et al., 2018; Gallage and Gil, 2014; Jeffries et al., 2019). The ciliated DEGAs in the 16 brain areas largely encoded the basal body and axonal components (Chen et al., 2021). The majority of DEGA in the axonal, basal, and transition regions is upregulated with age, and most of the genes encoding centrosome components are downregulated with age (Alvarado et al., 2021). Genetic mutations can cause changes in the structure and function of PC and lead to a range of clinical outcomes, including various types of encephalopathis (Boonsawat et al., 2019; Brancati et al., 2010; Robinson et al., 2020). Aging disrupts the central nervous system (CNS) and causes structural and functional changes in neural stem cells (NSC) and their niche, thereby affecting neural development and brain homeostasis, leading to decreased brain function (Capilla-Gonzalez et al., 2015; Fuentealba et al., 2012). Emerging data suggest the role of PC in the pathophysiology of neurodegenerative diseases (AD, PD, etc.), and confirm that changes in primary ciliary structure are closely associated with aging and many neurodegenerative diseases (Morelli et al., 2017; Bae et al., 2019). PC have been shown to contribute to PD in many aspects, such as signaling pathways, mitochondrial dysfunction, genetic variation, and dopaminergic neuronal changes in the substantia nigra (Dhekne et al., 2023; Ignatenko et al., 2023; Marchetti, 2018; Wang et al., 2022; Turcato et al., 2022). This review will summarize the systemic role of PC in the development of PD.

## 2 Changes of structure and formation of PC in PD

The two primary cell types in the brain are neurons and glial cells. The brain’s glial cells comprise oligodendrocytes, microglia, ependymal cells, and astrocytes (Xingi et al., 2023). There is evidence that microglia and mature oligodendrocytes have PC (Guemez-Gamboa et al., 2014). PC are also found in adult neural stem cells (or astrocytic type 1 radial glia) in the subgranular zone (SGZ) and the sub-ventricular zone (SVZ) of the lateral gyrus, which are essential for adult neurogenesis (Ming and Song, 2011; Han et al., 2008; Tong et al., 2014). An *in vivo* systemic study showed that genetic alterations associated with ciliopathy influence the formation of neural progenitor cells, early neural connection, neuronal differentiation, and neuronal radial migration during cortical development, which are critical for adult neurogenesis and exhibit neurodegenerative changes (Park et al., 2019; Guo et al., 2015).

One of the main risk factors for neurodegenerative illnesses is thought to be aging. Age-related disorders, such as PD, have been linked to PC and ciliary signaling (Zhao et al., 2018). It was found that the expression of mitochondrial DEGA, individual BBSomes DEGA, α-tubulin (mainly TUBA1A), and β-tubulin (TUBB2A, TUBB2B, and TUBB 3) were all down-regulated with age. γ-Tubulin complex-associated proteins (TUBGCP3, TUBGCP4, TUBGCP5, and TUBGCP6) (Miller et al., 2014; Spalding et al., 2013; Pedersen and Rosenbaum, 2008; Mourao et al., 2016; Hirano et al., 2017; Schroter et al., 2021; Aiken et al., 2020; Labisso et al., 2018). These microtubules are dynamic cytoskeletal polymers and vital parts of the PC, and they are involved in cellular activities that are vital to the development of the brain, such as neuronal migration, proliferation, and cortical laminar organization (Kumamoto et al., 2012; Minoura, 2017). Abnormal accumulation of TUB is a pathological feature of progressive supranuclear palsy (PSP) and cortical basal ganglia degeneration (CBD) (Pantelyat, 2022). This suggests that the absence of α-tubulin and β-tubulin may indicate that natural brain aging contributes to the development of neurodegenerative diseases. The accumulation of γ-tubulin is closely related to the typical clinical manifestations of PD. Some scholars have used acute knockout of the ciliary gene in the developing cortex (Kif 3 a or Ift 88) to study the effect of PC on cell-autonomous function in neural stem cells. This causes neuronal differentiation and migration defects, accompanied by a delay in the cycle progression of neural stem cells and a failure of nuclear migration between movements (Chen et al., 2019).

PC are important organelles transduced by Sonic Hedgehog (SHH) signaling, and their basal bodies contain components of Wnt and Notch signaling pathways (Rimkus et al., 2016). Researchers have found that Wnt signaling activity and Notch signaling activity associated with PC decreased significantly, and SHH signaling and TGFβ signaling activity increased with age (Kumamoto et al., 2012; Inestrosa et al., 2020; Serrano et al., 2014; Rivera et al., 2016; Sun et al., 2013; Kandasamy et al., 2014). Schmidt et al. found that PD mice have increased SHH-signaling, decreased nucleus levels of GLI3-FL and GLI3-R in the nucleus, and substantial dysregulation of cilia-associated potential target genes, including SHH (Schmidt et al., 2022). Furthermore, IFT complexes (IFT-A and IFT-B) construct and maintain PC and are necessary for SHH pathway activity (Qin et al., 2011). The deletion of the IFT-A genes results in minor abnormalities in the PC’s architecture and ectopic activation of the SHH pathway (Mukhopadhyay et al., 2010). Increased SHH signaling is found in a mouse model with a partial deletion of IFT-A (Liem et al., 2012). Various mutants (e.g., IFT 88, DYNC2H1, and KIF3A knockout) are also seen in PD mice, resulting in the disruption of the primary ciliary signaling mechanism (Wen et al., 2010). Wnt signaling impairment is linked to the degeneration and death of dopaminergic neurons in the substantia nigra midbrain in PD (Stephano et al., 2018). The dysregulation of serotonin signaling (HTR4/6/7 signaling) and DA signaling (DRD1/5 signaling) is an important mechanism of PD. Dysregulation of these pathways is accompanied by an enhancement of obviously changed G protein-coupled receptor (GPCR) signaling pathways (Huot et al., 2011; Hisahara and Shimohama, 2011). GPCRs are often localized on the neuroPC, and the ciliary output and downstream signal transduction components of activated GPCRs (GNAS, ADCY 2, ADPKs, and some MAPKs) are regulated by BBSome (Wheway et al., 2018; Ye et al., 2018). A protein transport complex called BBSome identifies transmembrane proteins with ciliary targeting sequences (such as class A and class B GPCR or RTK) and directs their combination to the IFT complex, which then transports the proteins to the ciliary membrane (Singh et al., 2020; Christensen et al., 2017). Thus, regulating BBSome within the cilium can regulate GPCRs and ameliorate the dysregulation of serotonin signaling and dopamine signaling, thereby improving PD. These studies suggest that the structural function of PC plays a crucial role in the PD disease process and warrant further investigation.

## 3 PC influence PD by signaling pathways

PC are essential in the signaling pathway for growth and development, disrupting PC by disrupting Hedgehog (HH) signaling, leading to developmental defects (Huangfu et al., 2003). Numerous studies have demonstrated that PC is impacted by the HH signaling pathway, which is crucial in controlling various facets of animal growth and development (Briscoe and Therond, 2013; Bangs and Anderson, 2017). One of the three HH proteins—Desert Hedgehog (DHH), Indian Hedgehog, or Sonic Hedgehog (SHH)—activates canonical HH signaling. Without HH ligands, Patched 1 is located in the PC, which prevents ciliary accumulation and the activation of G protein-coupled receptor proteins in SMO (Rohatgi et al., 2007), therefore suppressing HH signaling. Patched 1 leaves the PC after binding to HH, which causes its internal SMO to accumulate and activate (Corbit et al., 2005). Trigger SMO induces GLI 2 and GLI 3 transcription factors that stimulate the production of GLI transcriptional activators via HH signaling (Haycraft et al., 2005). When HH ligands are not present, GLI 2 and GLI 3 are cleaved into transcriptional repressors that stop the production of HH target genes. In the absence of HH, PC participation is also necessary for the formation of the GLI repressor protein (Huangfu and Anderson, 2005; Liu et al., 2005; May et al., 2005). Through controlling the ventral customizable, proliferative, and differentiated capabilities of precursor cells in the formation of the vertebrate central nervous system, SHH regulates the size, shape, and cell type of the brain (Ni et al., 2020). In addition, the SHH signaling pathway mediates the growth of intermediate precursor cells and radial glial cells to preserve neocortex neurons’ ability to proliferate, differentiate, and survive (Komada, 2012). In neural precursor cells, SHH and basic fibroblast growth factor 8 (FGF-8) synergistically act to promote the expression of genes involved in the formation of dopamine neurons (Britto et al., 2002), which is tightly associated with the genetic risk for PD (Agarwal et al., 2020). According to numerous researches, SHH can induce the differentiation of Bergmann glial cells (Wang and Almazan, 2016), and mediate the producion of oligodendrocyte precursors (Namchaiw et al., 2019; Starikov and Kottmann, 2020). An experiment with the chemical inhibitor or anti-SHH antibody made by Yang et al. (2020) showed that inhibiting SHH may inhibit oligodendrocyte differentiation. SHH dysregulation can contribute to developmental disorders. It has been found that the deregulation of the SMO-SHH signaling pathway can cause neurodegenerative diseases such as PD, nerve damage, neuronal excitotoxicity, increased oxidative stress, neuroinflammation, and apoptosis. The PI3K/Akt pathway is thought to be regulated by the SMO-SHH-GLI pathway, which in turn has neuroprotective, anti-inflammatory, and antioxidant effects (Prajapati et al., 2023; Shao et al., 2017). It has been shown that the loss of SHH gene damage occurs in differentiated DA neurons (Turcato et al., 2022). Intracerebral injection of SHH-N enhanced tyrosine hydroxylase immunoreactive neuronal expression in the striatum and improved motor performance in PD rats, indicating a specific treatment for PD (Yang et al., 2021). Therefore, PC has an effect on the pathology of PD by mediating the SHH pathway, and restoring the normal function of the SHH pathway is considered a critical target of PD treatment.

Another important regulator of animal development is Wnt signaling. Many of the signal transduction proteins acting on the Wnt pathway are located on the PC, including curprotein (FZD) and glycogen synthase kinase 3 (GSK 3) (Thoma et al., 2007; Veland et al., 2013; Stypulkowski et al., 2021). It contains ciliary-localized low-density lipoprotein receptor-associated protein (LRP 6) coreceptors that can respond to and transmit Wnt signals (Niehrs, 2012; Jeong and Jho, 2021). Wnt ligands are bound by LRP 6 and FZD transmembrane receptors, which then trigger a cytoplasmic signaling cascade that stabilizes the transcriptional coactivator β-catenin by blocking a destruction complex that contains GSK 3. GSK 3 is essential for normal embryonic development and is present in practically every tissue and organ system of an organism throughout its life (Kikuchi et al., 2011; Garcia de Herreros and Dunach, 2019). Furthermore, classical Wnt signaling, as expressed by β-catenin, but not classical signaling independent of β-catenin, mainly controls the cytoskeleton involved in planar cell polarity (Youn and Han, 2018). Cytoplasmic β-catenin is dephosphorylated when GSK 3 is inhibited. This occurs when proteins are freed from proteasomal degradation, brought into the nucleus, and bound by the transcriptional complex to control downstream target genes (Nusse and Clevers, 2017; MacDonald and He, 2012; Holzer et al., 2012). It is clear that PC are Wnt-transduced organelles that signal through the Wnt/GSK 3 signaling mechanism. The Wnt/β-catenin (WβC) pathway is known to be an important player in mDA-ergic neurogenesis and influence PD progression, which proceeds through classical transmembrane signaling across the plasma membrane (Munoz-Soriano and Paricio, 2011; Kallsen et al., 2015). Aging and PD are characterized by a reduction in the number of adult nerves, which is associated with the block of NSC activation to produce new neurons (Apple et al., 2017; Bond et al., 2015; Chandel et al., 2016). WβC has the ability to attach itself to transcription factors T cell factor and lymphoid enhancer binding factor (TCF/LEF), which in turn can stimulate the transcription of Wnt target genes that are important in the survival, proliferation, and differentiation of neural stem and progenitor cells (NSCs) (Brodski et al., 2019; Piccin and Morshead, 2011; Kalani et al., 2008; Adachi et al., 2007). There are only two specific regions in the dentate gyrus of the hippocampus (SVZ and SGZ) of the NSC that can generate new neurons under physiological conditions. These regions may be involved in odor discrimination, spatial learning, and contextual memory functions (Fuentealba et al., 2012; Spalding et al., 2013). Both regions have a large neural distribution of PC, structural defects in PC with increasing age, and early SVZ disruption and hippocampal SGZ neurogenesis are linked to premotor symptoms associated with PD (Agnihotri et al., 2019; Lim et al., 2018; Titova et al., 2017; Winner and Winkler, 2015). WβC signaling positively regulates adult neurogenesis (activation of stem cells from neuronal differentiation) at multiple levels, thereby improving PD symptoms (Ortiz-Matamoros et al., 2013; Varela-Nallar and Inestrosa, 2013; Hirota et al., 2016). Studies have shown that the potential of overcoming aging-induced loss of Wnt 1-mediated mNSCs in Aq-PVR: direct W β C signaling activation in MPTP-induced mice causes Aq-PVR-Nurr1 and TH + - precursors, hence supporting the histopathology and functional recovery of dark DA neurons, and then improving the clinical manifestations of PD disease (L'Episcopo et al., 2014; L'Episcopo et al., 2018; Marchetti et al., 2020).

An extremely significant developmental route that has persisted throughout mammalian evolution is the Notch signaling pathway. Through facilitating cell-to-cell communication, it controls cell motility. Five Notch ligands, four transmembrane receptors, the Notch intracellular domain (NICD), and the transcription factor RBP-J make up the majority of the mammalian Notch signaling pathway (Liang et al., 2023). The Notch receptor is located on the PC (e.g., Notch1 on the axon and basal body, Notch3 on the PC membrane), and the γ-secretase component Presenilin-2 that catalyzes NICD cleavage is located in the basal body (Leitch et al., 2014; Cervantes et al., 2010). The opening of Notch signaling activation involves two key pathways: I γ-secretase-dependent cleavage; cytoplasmic NICD release upon binding of the II ligand receptor to Notch ligand on the cell membrane of neighboring cells (Nijjar et al., 2001; McCright et al., 2002; Kodama et al., 2004; Croquelois et al., 2005). After translocation to the nucleus, the NICD binds RBP-J κ and converts it from a transcriptional repressor protein to an activator, leading to the transcription of Notch target genes (Rohatgi et al., 2007; Geisler et al., 2008). Notch regulates cilia formation and ciliary body length. We demonstrate that in the absence of Notch signaling, shorter cilia are generated, whereas hyperactive signaling produces longer cilia (Lopes et al., 2010). Notably, one study also showed that the LRRK2 complex in the mouse brain suppresses the expression of Notch signaling to accelerate neuronal differentiation (Imai et al., 2015). A recent study demonstrated that these mice suppressed the Notch signaling pathway to alleviate the motor deficit in an MPTP-induced mouse PD model (Wang et al., 2019). Many researches have found that the recombinant signaling binding protein J κ (RBP-J) can regulate Notch signaling, regardless of monocyte differentiation and macrophage activation under physiological or pathological conditions (Borggrefe and Oswald, 2009; Gamrekelashvili et al., 2016; Gamrekelashvili et al., 2020). Furthermore, inhibition of DA neuronal death and improved locomotor behavior in mice was achieved by myeloid-specific blockade of Notch signaling to lessen IM infiltration and MHCII+ microglial activation. By lowering the release of inflammatory cytokines, myeloid-specific RBP-J deficiency can slow the course of PD (Liang et al., 2023).

PD is characterized by the presence of Lewy neurites and intraneuronal Lewy bodies, which are in line with the development of symptom severity in different brain regions (Kempster et al., 2010; Braak et al., 2005; Saito et al., 2004). A significant decrease in dopamine (DA) levels is caused by the selective and gradual degeneration of midbrain dopaminergic (mDAergic) neurons in the dense substantia nigra (substantia nigra pars compacta, SNpc) and their projection to the striatum (L'Episcopo et al., 2014; L'Episcopo et al., 2018; Marchetti et al., 2020). The final diagnosis of PD is thought to be based on the presence of Lewy bodies in the substantia nigra (SN) and the loss of DA neurons (Hughes et al., 1992). Lewy bodies mostly consist of α-synuclein (α-syn) (Eriksen et al., 2003), which is highly stained in samples from PD patients (Shults, 2006). Thus, another characteristic of the pathophysiology of PD is the increasing accumulation of misfolded α-syn in cortical and subcortical brain areas (Trojanowski and Lee, 1998). Currently, the α -syn mouse model has been widely used in simulated PD pathology experiments, and existing studies have found that the Notch-1 expression is reduced in the α -syn Tg mouse model (Crews et al., 2008). α-syn inhibits Notch-1 transcriptional activity by decreasing the association of NICD and RBP-JK, as well as enhancing the interaction of NICD with Fbw 7, thereby increasing NICD degradation (Naushad et al., 2021; Wakabayashi, 2020). Moreover, an animal study also showed that continuous accumulation of α-syn causes reduced Notch-1 expression and its signaling, leading to decreased neural precursor (NPC) survival (Crews et al., 2008). Furthermore, α-syn aggregates in catecholaminergic neurons in PD are considered an impact factor for reduced neuronal health and ultimately cell death. Studies have confirmed that α-Syn aggregates have spread in the body like prions (PrP) (Chen et al., 2022). In the pathological state, α-Syn accumulates and escapes, is internalized by neighboring cells, and directly induces normal α-Syn misfolding and aggregation, thus achieving the purpose of diffusion (Fields et al., 2019; Rocha et al., 2018). Thus, these findings suggest that preventing α-syn aggregation and stopping α-Syn transmission may be a therapeutic treatment for PD.

Studies using epidemiological methods have shown that individuals with PD have a lower incidence of most cancer types (Zhou et al., 2022). A number of pathogeneses linked to PD have also been linked to the onset and progression of cancer, including abnormal mitochondrial function, an aberrant oxidative stress response, sustained mitogenic signals, and so on. These findings indicated a possible function of Parkinson’s genes in the development and progression of cancer (Ejma et al., 2020; D'Amelio et al., 2009). Recent reports have shown that α-Syn is highly expressed in melanoma (Matsuo and Kamitani, 2010; Lee et al., 2013), while it is downregulated in lung adenocarcinoma, breast cancer, and bladder cancer, and its expression could significantly impact the prognosis of cancer patients (Zhou et al., 2022; Yan et al., 2018; Wu et al., 2022). The process known as the epithelial-mesenchymal transition (EMT) is defined by the epithelial cells’ acquisition of mesenchymal traits and their loss of epithelial properties (Thiery et al., 2009). According to recent studies, PC deficiency both initiates and intensifies EMT when it is in a resting state and when fibrotic signals like TGF-b are present (Alvarez-Satta et al., 2021). Zhou et al. (2022) have confirmed that α-Syn overexpression can reverse the EMT process and suppress cell viability. However, further experiments are needed to explore how α-Syn inhibits migration, invasion, and EMT.

**Figure 1:**
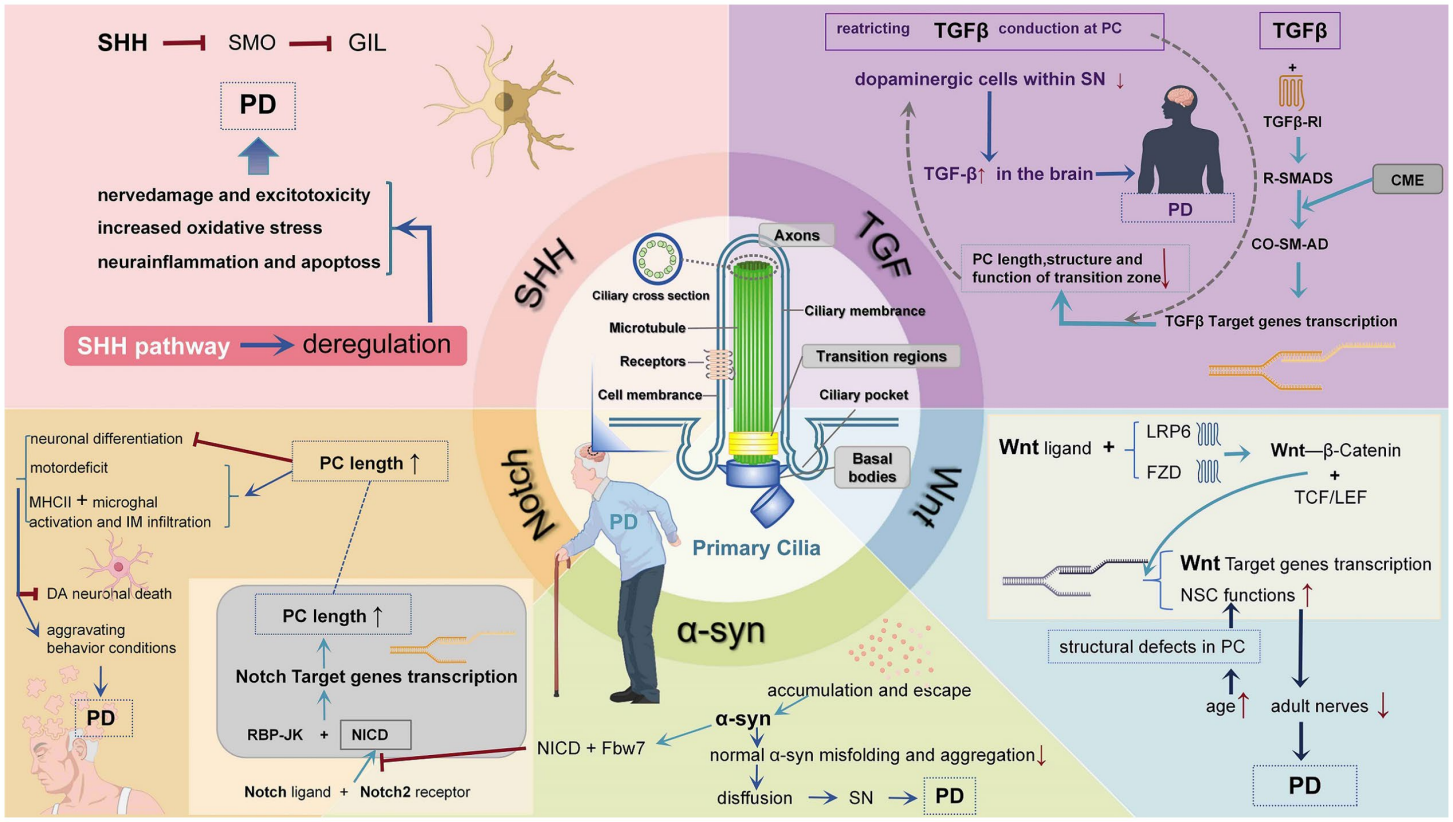
Mechanisms of PD that influenced by PC mutations. In various PD, PC plays an important role in mediating signaling pathways and maintaining neural cell homeostasis. Impaired PC function and structure affect pathways and transcriptional activity. (1) Patched 1 binding to HH in the SHH pathway promotes SMO accumulation to induce the production of GLI 2 and GLI 3 transcription factors. When HH ligand is not present, both ciliary accumulation and G protein-coupled receptor protein activation are blocked, and GLI 2 and GLI 3 are cleaved into transcriptional repressors, hindering HH target gene production, leading to PD. (2) TGF-β signaling in PC operates through activation of receptor R-SMAD transcription factors and internalization of active receptors through clathrin-mediated administration of the cilia pocket endocytosis (CME). R-SMAD binds to co-SMAD and initiates target gene transcription. Blocking TGF-β signaling causes cilia shortening and impairs the structure and/or function of the transition zone. Knocking out TGF-β signaling genes causes a decreased quantity of dopaminergic neurons in the SN, which raises the possibility of PD. (3) When the expression of Notch signaling is suppressed, DA neuronal death can be inhibited and locomotor behavior can be improved. α-syn inhibits Notch-1 transcriptional activity by decreasing the association of NICD and RBP-JK, as well as enhancing the interaction of NICD with Fbw 7, causing reduced Notch-1 expression and its signaling, leading to decreased neural precursor (NPC) survival. (4) α-Syn accumulates and escapes, is internalized by neighboring cells, and directly induces normal α-Syn misfolding and aggregation, thus achieving the purpose of diffusion and causing PD. (5) Wnt ligands bind to LRP 6 and FZD receptors, block the destruction complex containing GSK 3, and transcribe β-catenin. W β C binds to TCF and LEF and stimulates Wnt target gene transcription, thereby improving PD symptoms. Notch ligand bind to receptor and release cytoplasmic NICD, which binds RBP-J κ, leading to the transcription of Notch target genes.

The transforming growth factor β (TGF-β) growth factor superfamily includes TGF-β, activins, and BMP. Activated TGFβ ligands interact with type II TGF-β receptor (Tβ RII) and by TβRII aggregation and phosphorylates type I TGFβ receptor (Tβ RI) to activate downstream signaling via the SMAD receptor (Varela and Garcia-Rendueles, 2022; Kashima and Hata, 2018). TGF-β signaling in PC operates through activation of receptor R-SMAD transcription factors (SMAD 2/3 and SMAD 1/5/8) and internalization of active receptors through clathrin-mediated administration of the cilia pocket endocytosis (CME) (Garcia-Gonzalo and Reiter, 2017). R-SMAD forms a trimeric complex with co-SMAD SMAD4, which translocates into the nucleus and initiates target gene transcription. TGF-β also activates extracellular signal-regulated kinases (ERK1/2) in PC (Oh and Katsanis, 2013). Some cell experiments show that selectively restricting TGF-β conduction at the primary ciliary plasma membrane can inhibit the ciliary information transfer function and subsequently prevent cell migration and invasion (Clement et al., 2013; Labour et al., 2016; Alvarez-Satta et al., 2021). One study also showed that embryos from Tg737 and Ift88 null mice were deficient in PC formation with aberrant TGF-β signaling (Clement et al., 2009; Arthur and Bamforth, 2011). Besides, TGF-β signaling is critical to the function of the transition zone, which may in turn affect the regulation of ciliary length (Szymanska and Johnson, 2012). It has been indicated that blocking TGF-β signaling by injecting DN-S2 RNA and examining the ciliary morphology of the neural tube, revealed that blocking TGF-β signaling causes cilia shortening and impairs the structure and/or function of the transition zone (Tozser et al., 2015). Besides, activation of TGF-β receptors promotes SHH signaling, which creates even more ciliary signaling pathway crosstalk (Garcia-Gonzalo and Reiter, 2017). The TGF-β pathway controls important physiological processes in tissue, cell growth, development, and homeostasis in normal cells (David and Massague, 2018). The TGF-β superfamily plays a critical role in neuroinflammation and repair after brain injury (Kashima and Hata, 2018). Researches have indicated that TGF-β plays a critical part in age-dependent diseases (e. g., PD), and it increases in the brains of patients with PD (Vawter et al., 1996). A recent study showed that several mouse models knocking out TGF-β signaling genes showed a decreased number of dopaminergic cells within the SN, suggesting that impaired TGF-β signaling increases the risk of PD. Moreover, the study showed that loss of TGF-β signaling in neurons is an important marker of SN degeneration (Tesseur et al., 2017). These results suggest that the PC effect on TGF-β may act as a significant part in regulating the process of neurodegeneration seen in PD (Figure 1).

## 4 PC and dysfunction of mitochondrial

Functions and morphology of mitochondrial are highly affected by mitochondrial dynamics. Therefore, abnormalities in mitochondrial dynamics are related to lots of human diseases, including neurodegenerative diseases (Baek et al., 2017). It has been reported that PD pathogenesis is significantly influenced by impaired mitochondrial activity and the ensuing rise in oxidative stress, neuroinflammation, and microglial activation (Moon and Paek, 2015). The regulation of cellular adaptation to stresses, such as decreased biogenesis, aging, damage to mitochondrial DNA (mtDNA), nutritional limitation, and abnormal imbalances between fission and fusion events, is largely dependent on mitochondria. If these activities continue uncontrolled, ROS can harm proteins, lipids, and nucleic acids, leading to persistent oxidative stress (Youle and van der Bliek, 2012; Pickles et al., 2018). The dynamics of mitochondria are influenced by oxidative stress via posttranscriptional changes such as ubiquitination (Wu et al., 2010). Consequently, damaged mitochondria accumulate, which ultimately results in cell death and more general tissue malfunction. The heart, muscles, brain, and retina are among the tissues with high energy demands that are most susceptible to mitochondrial dysfunction (Wallace et al., 2010).

Autophagy is a catabolic process, which is highly sensitive to extracellular and intracellular stress. Through cellular energy balance regulation and the facilitation of organelle quality control, it preserves cellular homeostasis (Mizushima, 2018). Numerous evidence have indicated a close reciprocal relationship between PC and autophagy, as do autophagy and mitochondrial dynamics/functions (Yoo and Jung, 2018; Palikaras et al., 2018; Wiegering et al., 2019; Wang et al., 2015). PC regulate autophagy mutually, which provides protection by removing damaged mitochondria under oxidative stress conditions (Yoo and Jung, 2018; Wang et al., 2015; Ávalos et al., 2017). By means of IFT and Hh-dependent processes, autophagy-related proteins (ATGs) shuttle to the basal body and PC (Pampliega et al., 2013). Serum-deprived cells’ ability to undergo autophagy is suppressed by the interference with ciliogenesis and Hh signaling (Pampliega et al., 2013). On the other hand, autophagy eliminates IFT20 or oral-facial-digital syndrome 1 (OFD1), respectively, to suppress or mimic ciliogenesis (Pampliega et al., 2013; Tang et al., 2013).

A previous research proposed that autophagy driven by both mitochondrial reactive oxygen species (mtROS) and AMP-activated protein kinase (AMPK) are important pathways that underlie ciliogenesis triggered by mitochondrial stress (Bae et al., 2019). Damage of PC are proved to contribute to many neurological abnormalities. Gain-of-function mutations in somatic mechanistic target of rapamycin (mTOR) hinder ciliogenesis in the developing brain by compromising the autophagic clearance of OFD1, which results in localized malformations in cortical development (Park et al., 2018). Dysfunction of PC by loss of ciliary proteins such as PCM1 and Tctn3 caused neuronal apoptosis in mice (Wang et al., 2018), which is consistent with the pathological basis of PD.

The majority of PD biochemical and pathological hallmarks are produced by the mitochondrial neurotoxin 1-methyl-4-phenyl-1,2,3,6-tetrahydropyridine (MPTP). MPTP binds mainly in astrocyte lysosomes, where it is transformed by type B monoamine oxidase into 1-methyl-4-phenylpyridinium ion (MPP+), which subsequently causes a parkinsonian syndrome (Marini et al., 1992). By blocking complex I, MPP+ can hinder mitochondrial respiration (Nicklas et al., 1985; Trevor et al., 1987; Sayre et al., 1989; Przedborski and Jackson-Lewis, 1998). In addition, we have shown that in an MPTP-induced PD model and toxic PD models, PC were observed remarkable elongation, and increased primary ciliogenesis in DA neurons encourages autophagy as well as the survival of neurons (Bae et al., 2019; Miyoshi et al., 2014). In contrast, SN neurons with impaired ciliogenesis are more likely to undergo apoptosis and are unable to trigger autophagy (Bae et al., 2019).

PC are considered to have protective effects of neurons. Following an axotomy, PC can increase retinal ganglion cell survival by reducing oxidative stress (Choi et al., 2019). In a PD model, however, the inhibition of PC ciliogenesis exacerbated motor impairment and neuronal loss (Bae et al., 2019). Bae et al. (2022) discovered that the use of L-carnitine and acetyl-L-carnitine decreased the risk of mitochondrial fragmentation, ROS production and MPP + -induced neurotoxicity in MPP + -treated cells. It promoted ciliogenesis of PC in the SH-SY5Y cells, which helps to protect neurons from oxidative stress and cell death brought on by MPP+. In healthy mice, acetyl-L-carnitine given over an extended period of time can enhance brain energy levels (Scafidi et al., 2010; Smeland et al., 2012). In contrast, the inhibition of PC caused by knockout intraflagellar transport 88 (IFT88) totally reversed the protective effect of L-carnitine and acetyl-L-carnitine against MPP + -induced cell death (Bae et al., 2022).

MPTP-induced DAergic neurotoxicity has been proposed to be significantly influenced by the mitochondrial apoptotic cascade (Mochizuki et al., 2001; Viswanath et al., 2001). In addition to causing neurotoxicity, MPTP also causes an inflammatory response, which includes T cell infiltration into the SN and striatum, activation of resident brain macrophages and microglia, and elevated proinflammatory cytokine gene expression (Kurkowska-Jastrzebska et al., 2009). In addition, active microglia can generate pro-inflammatory factors that are toxic to neurons and to be phagocytic (Banati et al., 1993). Of note, neuroinflammation is considered as a vital marker of the pathogenesis and development of neurodegenerative diseases (including PD) (Zhang et al., 2024).

According to Belin and Westerlund (2008), Parkin (GeneID: 5071) and PINK1 (GeneID: 65018) gene mutations are the most common cause of early-onset PD. Familial variants of PD206 are linked to mutations in the Parkin RBR E3 ubiquitin-protein ligase (PARKIN) and PTEN-induced kinase 1 (PINK1) genes (Quinn et al., 2020). Previous researches considered PINK1 acting as a pivotal neuroprotective protein, which contribute to prevent mitochondrial dysfunction and activate basal and starvation-induced autophagy (Gautier et al., 2008; Gelmetti et al., 2008; Wood-Kaczmar et al., 2008; Levine et al., 2008), by its interaction with Beclin-1, a key pro-autophagic protein that has previously correlation to the pathogenesis of several neurodegenerative illnesses (Michiorri et al., 2010). Thus, treatment approaches that target PINK1/PARKIN signaling may be able to treat PD.

Researches from Schmidt et al. (2022) displayed dysregulated respective genes and dysfunction of intraflagellar transport (IFT) as well as IFT interaction with the BBSome in early sporadic PD patients (Das Banerjee et al., 2017). The enrichment of markedly changed G protein-coupled receptors (GPCR) signaling pathways, such as dopamine (DRD1/5 signaling) (Hisahara and Shimohama, 2011) and serotonin (HTR4/6/7 signaling) (Huot et al., 2011), which are both linked to PD, coincides with a dysregulation of these pathways. They made an objective molecular characterization of hiPSC-derived neuronal precursor cells (hNPCs) using multiplexed, droplet-based, single-cell RNA sequencing (scRNA-seq). This method revealed a large number of genes linked to PC function in both the cellular model and the postmortem tissue of individuals with sPD. The roles and locations of these genes inside the cilia allow them to be categorized into many groups, including ciliogenesis (e.g., INTU, CEP83, OCRL), axoneme (TUBA1A, TUBB), transition zone (e.g., AHI1), and intraflagellar transport (IFT: e.g., IFT74, IFT52, IFT20, DYNC2H1, 61). Moreover, they elucidated that PC dysfunction is the basis of mitochondrial respiratory deficiencies and ciliary-mediated SHH-signaling, which is also a common molecular characteristic of early PD, including sporadicand familial (PINK1, LRRK2) PD (Schmidt et al., 2022).

Taken together, PC probably play a potential role in PD pathology by the interplay with autophagy and the relationship between autophagy and mitochondrial dynamics/functions, of which are worthy to be explored further.

## 5 PC and genes

Non-muscle myosin II (NMII) are major regulators of actin cytoskeletal dynamics, which play a critical role in cellular organization, polarity, morphology, migration and division etc. Altered NMII was found in the complex pathologies that cause numerous neurological disorders (Nadif Kasri and Van Aelst, 2008; Newell-Litwa et al., 2015), including neurodevelopmental disorders, neurodegenerative disorders, neuronal migration disorders (Tsai et al., 2007). The NMII heavy chains (NMHCII) play a critical role in dimerization and other interactions (Ronen and Ravid, 2009). Variants of NMHCII genes are associated with numerous human diseases, for example, a broad spectrum of neurodevelopmental disorders caused by MYH10 (Kopinke et al., 2021). Two reports demonstrated that MYH10 is implicated in the biogenesis of PC, and the knockdown of MYH10 siRNA result in a profound defect in PC (Rao et al., 2014; Hong et al., 2015). Moreover, neuronal migration defects, such as corpus callosum defects, and cerebellar hypoplasia etc. have been detected in mouse models and patients associated with alterations in PC and the Hedgehog signaling pathway (Kopinke et al., 2021; Elliott and Brugmann, 2019). A previous experiment demonstrated that MYH10 knockout cells show shortened ciliary length along with defective Hedgehog signaling and abnormalities in PC ciliogenesis. The overexpression of MYH10 variant resulted in a dominant-negative influence on ciliary length (Kopinke et al., 2021).

In clinic, NMII kinases are considered as promising targets for the treatment of diverse synaptopathies. Rho-kinase (ROCK) has been considered to activate NMII (Kaibuchi et al., 1999; Leung et al., 1996), and it has been demonstrated that ROCK inhibitors, which encourage neurite outgrowth to reestablish neural connections, are effective in treating PD (Lopez-Lopez et al., 2023). Conditions resulting from abnormal NMII activity are currently treated using vasodilators, which target myosin to modulate blood pressure. A vasodilator called Fasudil has been successfully used to improve neuron survival and motor function in PD rodent models (Tönges et al., 2014; Zhao et al., 2015).

It has been generally accepted that leucine-rich repeat kinase 2 (LRRK2) mutations are a cause of inherited PD (Paisán-Ruíz et al., 2004; Zimprich et al., 2004), and the LRRK2 protein are one of the central molecules in PD researches (Cookson, 2015; Domingos et al., 2019; Lee et al., 2012). The Rab GTPase family is an essential component of the LRRK2 signaling pathway as it acts as a kinase substrate and LRRK2 regulator (Purlyte et al., 2018; Steger et al., 2017; Steger et al., 2016; Taylor and Alessi, 2020), especially Rab10 and Rab8A (Yeshaw et al., 2023). Intracellular vesicle trafficking is regulated by membrane-anchored Rab proteins (Homma et al., 2021). There have been reports of defective vesicle trafficking in cells harboring PD-causative LRRK2 mutations and considered as a culprit of PD (Gao et al., 2018). At the same time, pathogenic LRRK2 kinase can interfere with ciliogenesis. The failure of LRRK2-generated phosphoRab10-RILPL1 and phosphoRab10-MyoVa complexes to recruit TTBK2 to the mother centriole and to interact with CEP164 to inhibit PC formation (Dhekne et al., 2021; Sobu et al., 2021). Pathogenic LRRK2 also enhances PC loss by a yet-to-be-determined, Rab10 and RILPL1-independent pathway (Sobu et al., 2021). Berndsen et al. (2019) and Khan et al. (2021) discovered loss of protein phosphatase 1H (PPM1H) phenocopies hyperactivation of LRRK2 in cell culture and mouse brain, and considered Rab-specific phosphatase PPM1H has the ability to reverse LRRK2-mediated Rab phosphorylation. In addition, PC production in wild-type (WT) cells can be inhibited by depleting PPM1H phosphatase (Berndsen et al., 2019), of which the phenotype is similar to that of PC loss upon expression of pathogenic LRRK2 (Berndsen et al., 2019). Previous researches found that the PC in rare, striatal, cholinergic interneurons decrease in mice carrying the R1441C LRRK2 mutation, which would impair these cells’ capacity to perceive Hh signals (Dhekne et al., 2018).

## 6 PC and substantia nigra dopaminergic neurons

In neurons, PC represents an electrical micro-domain (Jiang et al., 2019; DeCaen et al., 2013; Pablo et al., 2017). PC in differentiated cells are considered to be responsive to variations in glucose content. A previous experiment showed an inhibitory impact on the firing of substantia nigra (SN) dopaminergic (DA) neurons in high glucose (Saller and Chiodo, 1980). SN DA neurons have been discovered progressively lost in PD patients with type II diabetes, which seems that type II diabetes increase the risk of PD (Sportelli et al., 2020; Hassan et al., 2020). Several studies indicate that PC play a significant role in DA signaling transmission (Domire et al., 2011; Leaf and Von Zastrow, 2015). The surface of PC of different cell types is occupied by G-protein-coupled receptors, excitatory D1-type dopamine receptors (D1Rs) (Domire et al., 2011), which are transported to PC from the extraciliary plasma membrane by the intraflagellar transport complex-B (IFT-B) (Domire et al., 2011; Leaf and Von Zastrow, 2015; Marley and von Zastrow, 2010). The DA midbrain neurons are the source of the DA input. PC in the striatum is elongated in a number of pharmacological and genetic models of PD due to DA input deficits (Bae et al., 2019; Miyoshi et al., 2014; Steger et al., 2017; Dhekne et al., 2018; Jiang et al., 2019; Lucarelli et al., 2019). Longer PC has been hypothesized to be more sensitive in chemo- or mechano-sensation (Wang and Dong, 2013; Boehlke et al., 2010). When PC is lost, the proportion of DA-excited “active” SN neurons decreased, while that of DA-inhibited “desensitized” SN DA neurons increased (Mustafa et al., 2021). Thus, PC are indispensable for preserving the integrity of the nigrostriatal system and SN DA neurons (Table 1).

**Table 1:** Summary of mechanisms in autophagy, genes and SNDA neurons that PC influenced and the therapeutic attempts of Parkinson’s diseases.

Mechanism	Factor	Alteration of PC	Therapeutic attempt	References
Autophagy	mtROS AMPK	Autophagy driven by both mitochondrial reactive oxygen species (mtROS) and AMP-activated protein kinase (AMPK) are important pathways that underlie ciliogenesis triggered by mitochondrial stress	Not available	Bae et al. (2019)
	mTOR OFD1	Gain-of-function mutations in somatic mechanistic target of rapamycin (mTOR) hinder ciliogenesis in the developing brain by compromising the autophagic clearance of OFD1.	Not available	Park et al. (2018)
	MPP+	PC were observed remarkable elongation in an MPTP-induced PD model and toxic PD models.	Ji-Eun Bae et al. discovered that the use of L-carnitine and acetyl-L-carnitine decreased the risk of mitochondrial fragmentation, ROS production and MPP + -induced neurotoxicity in MPP + -treated cells. It promoted ciliogenesis of PC in the SH-SY5Y cells, which helps to protect neurons from oxidative stress and cell death brought on by MPP+.	Bae et al. (2019) and Miyoshi et al. (2014)
Genes	Ciliogenesis (INTU, CEP83, OCRL), axoneme (TUBA1A, TUBB), transition zone (AHI1), and intraflagellar transport (IFT: IFT74, IFT52, IFT20, DYNC2H1)	Not available	Not available	Schmidt et al. (2022)
	PINK1	As a result of the alterations in intraflagellar transport the PINK1-deficient hNPC populations exhibited significantly shorter PC.	Not available	Schmidt et al. (2022)
	NMII, MYH10	MYH10 knockout cells show shortened ciliary length along with defective Hedgehog signaling and abnormalities in PC ciliogenesis.	Rho-kinase (ROCK) has been considered to activate NMII, while ROCK inhibitors, which encourage neurite outgrowth to reestablish neural connections, are effective in treating PD. Conditions resulting from abnormal NMII activity are currently treated using vasodilators, which target myosin to modulate blood pressure. A vasodilator called Fasudil has been successfully used to improve neuron survival and motor function in PD rodent models.	Rao et al. (2014), Hong et al. (2015), Kaibuchi et al. (1999), Leung et al. (1996), Lopez-Lopez et al. (2023), Tönges et al. (2014), and Zhao et al. (2015)
	LRRK2	The failure of LRRK2-generated phosphoRab10-RILPL1 and phosphoRab10-MyoVa complexes to recruit TTBK2 to the mother centriole and to interact with CEP164 to inhibit PC formation. Pathogenic LRRK2 also enhances PC loss by a yet-to-be-determined, Rab10 and RILPL1-independent pathway.	Loss of protein phosphatase 1H (PPM1H) phenocopies hyperactivation of LRRK2 in cell culture and mouse brain, and Rab-specific phosphatase PPM1H has the ability to reverse LRRK2-mediated Rab phosphorylation.	Dhekne et al. (2021), Sobu et al. (2021), Berndsen et al. (2019), and Khan et al. (2021)
SN DA neurons	G-protein-coupled receptors, D1Rs, IFT-B	PC in the striatum is elongated in a number of pharmacological and genetic models of PD due to DA input deficits.	Not available	Bae et al. (2019), Miyoshi et al. (2014), Steger et al. (2017), Dhekne et al. (2018), Jiang et al. (2019), Domire et al. (2011), Leaf and Von Zastrow (2015), Marley and von Zastrow (2010), and Lucarelli et al. (2019)

## 7 Conclusion

Based on the exposition described above, dysregulation of neuronal PC make a significant effect on mediating signaling pathways and maintaining homeostasis of neuron cells in PD. In SHH pathway, the deregulation of SMO-SHH signaling pathway can cause neurodegenerative diseases such as PD, and the activation of the SMO-SHH-GLI pathway has neuroprotective, anti-inflammatory and antioxidant properties. On the other hand, the SHH pathway buffers dopaminergic neurons against oxidative stress by increasing the activity of SOD1 as well. In Wnt pathway, it is supposed that PC are Wnt-transduced organelles that signal through the Wnt/GSK 3 signaling mechanism. Direct W β C signaling activation in MPTP-induced mice causes Aq-PVR-Nurr1/TH + −precursors, thus are conducive to the histopathology and functional recovery of dark DA neurons, and then improving the clinical manifestations of PD disease. LRRK2 complex suppresses the expression of Notch signaling to reduce inflammatory cytokine secretion and alleviate the motor deficit. At the same time, continuous accumulation of α -syn causes reduced Notch-1 expression and its signaling, leading to decreased neural precursors (NPC) survival. An additional layer of communication between the ciliary signaling pathways is added when TGF β receptors are activated, which stimulates SHH signaling. There is a close relationship between PC and autophagy, as do autophagy and mitochondrial dynamics/functions. Autophagy driven by both mitochondrial reactive oxygen species (mtROS) and AMP-activated protein kinase (AMPK) are major mechanisms underlying mitochondrial stress-induced ciliogenesis. Damage of PC are proved to contribute to many neurological abnormalities, such as caused neuronal apoptosis. MYH10 is implicated in the biogenesis of PC, and the knockdown of MYH10 siRNA result in a profound defect in PC, which influence HH pathway. LRRK2 inhibit PC formation and contribute to PD via defective vesicle trafficking. PC are indispensable for the preservation of SN DA neurons and the integrity of nigrostriatal. However, genetic ablation of SHH in differentiated DA neurons results in their degeneration. Whether PC contribute to PD by influencing SHH pathway is still uncertain.

Currently, a number of studies have provided a solid scientific foundation for clinical research and the treatment of PD. These studies have revealed the mechanism of the role that PC play in neurodegenerative diseases from a microscopic perspective, including signaling pathways, genes, defective mitochondrial function, and substantia nigra dopaminergic neurons. Research has suggested that astrocytes and other cell types may possibly play a role in many neurological conditions, such as AD and PD (Joe et al., 2018; Sanchez et al., 2021; Verkhratsky et al., 2010; Vincent et al., 2010). An experiment conducted by Sterpka and Chen (2018), who are focusing on visualization of PC in mice astrocytes, detected a high expression of AC3 and high levels of ARL13B expression in the PC of astrocytes 10 days after birth and postnatal day 56, respectively. Transgenic mice lacking PC showed abnormal development and disruption in Shh signaling in astrocyte-like neural precursors (Breunig et al., 2008). And ARL13B disruption in PC can lead to decreased Shh signaling in mouse medulloblastoma cell cultures (Bay et al., 2018). Nevertheless, little is known about the function of PC and ciliary signaling in astrocytes during these illnesses to far. This may be due to the fact that PC in astrocyte in the mature brain are typically understudied, and the Shh pathway in neuronal PC is more significant during neurodevelopment than it is throughout maturity. As for Notch pathway, the decrease of Notch signaling makes PC shorter and will slow down the symptoms of PD. While α-syn would lower Notch-1 expression, A-syn would accumulate and cause PD. Contrary to the aforementioned experimental findings, PC length strain is short and PD symptoms should slow down if there is no other change at this period. We hypothesize that they will probably operate in situations where space and time are dependent on one another. These findings imply that α-syn has a role in altering the PC structural function of brain cells, although more research is required to determine the precise process. Furthermore, there is little data on the longevity and development of PC in different neurodegenerative disorders. It would be worthwhile to carry out more research to clarify the connection between PC’s morphological structure and function.

In addition, although there are a few studies on the treatment of PD, such as LiCl activating Wnt pathway conduction (Qi et al., 2017), bromocriptine activating D2 receptor (Miyoshi et al., 2014), and MLi-2 inhibiting LRRK2 kinase activity (Steger et al., 2017), etc., they rarely mention whether there is any change in the structure and function of PC in the process of pathway change. There is also a lack of specific biomarkers to measure the level of inhibition or activation of enzymes that affect pathways or gene expression, as well as the targets involved in treatment. However, AD has been shown to treat AD with 5-HT6 antagonists SB271046 reduction of cilia length (Hu et al., 2017). Targeting Shh signaling, such as bone marrow stromal cells, BMSCs activate Shh/Gli1 signaling to promote oligodendrocyte production, which in turn treats stroke (Zhang et al., 2009). Both L-carnitine and L-carnitine induce ciliary production, which protects nerves from oxidative stress and cell death (Bae et al., 2022). Therefore, it was hypothesized that antagonizing 5-HT6 receptors to reduce cilia length, targeting Shh/Gli1 signaling to alleviate PC defects and oligodendrocyte synaptic injury, and using L-carnitine and L-carnitine to promote PC production to protect nerves could alleviate PD symptoms. If this hypothesis can be applied to future research to test its feasibility, it is expected to become a new treatment method after passing clinical trials. It is hoped that significant progress will be made in the treatment of PD in the near future.
